# Progression Free Survival and Predictor of Recurrence in DLBCL patients with Negative Interim ^18^FDG PET/CT Using Standardized Imaging and Reporting Protocols

**DOI:** 10.31557/APJCP.2020.21.8.2343

**Published:** 2020-08

**Authors:** Maseeh uz Zaman, Nosheen Fatima, Areeba Zaman, Unaiza Zaman, Sidra Zaman, Rabia Tahseen

**Affiliations:** 1 *Department of Radiology, Aga Khan University Hospital, Karachi, Pakistan. *; 2 *Department of Medicine, Dr Ruth Pfau Hospital, Karachi, Pakistan. *; 3 *Department of Medicine, SUNY Downstate, Hospital, New York, USA. *; 4 *Dow Medical College, Dow University of Health Sciences (DUHS), Karachi, Pakistan. *; 5 *Department of Radiation Oncology, Aga Khan University Hospital, Karachi, Pakistan. *

**Keywords:** Interim PET/CT, DLBCL, negative iPET, lymphoma, recurrence, progression free survival, predictor

## Abstract

**Background:**

To determine progression free survival (PFS) and predictor of recurrence in patients with diffuse large B-cell lymphoma (DLBCL) with negative interim ^18^FDG PET/CT (iPET) using standardized imaging and reporting protocols.

**Materials and Methods:**

This prospective study was conducted at PET/CT Section of a JCIA accredited healthcare facility from December 2015 till February 2020. Patients with DLBCL having complete metabolic response (CMR; Deauville score: 1-3) on iPET were selected and followed for a median period of 11 months (4-144 months). End point response on follow-up PET/CT (either end of treatment or surveillance) was categorized as sustained CMR (sCMR) and disease recurrence. Kaplan Meier survival curve was used to measure PFS and receiver operating characteristics (ROC) was plotted for age, largest lesion size, highest standardized uptake value (SUVmax), disease stage and body mass index (BMI) on baseline scan to find their impact on recurrence.

**Results:**

Total 185 patients with DLBCL who had achieved CMR on iPET with a median age 55 years (19 – 88 yr.) with male predominance (63% male) were selected. On follow-up, 123 (66%) had sCMR while recurrence was found in 34% (p <0.05). No significant difference in demographics was found between two groups. Median PFS time was 34 months (22.8 – 45.1 months). On ROC analysis, only baseline highest SUVmax was found as a significant independent predictor of disease recurrence at a cut off >22.6 (highest area under curve: 0.595; SE 0.046; p <0.05).

**Conclusion:**

We conclude that recurrence is found in 34% of DLBCL patients with a negative interim ^18^FDG PET/CT using standardized imaging and reporting protocols. Despite of early response, these patients need continued intensive follow-up especially those with a baseline SUVmax > 22.6.

## Introduction

Diffuse large B-cell lymphoma (DLBCL) represents the most common subtype of adult Non-Hodgkin lymphoma (NHL) cases with an aggressive clinical course (Burggraaff et al., 2019). With standard rituximab plus cyclophosphamide, doxorubicin, vincristine and prednisone (R-CHOP) cure is achieved in 60-70% cases (Gisselbrecht et al., 2010). However, treatment failure is still an important problem as the 3-year progression-free survival (PFS) is approximately 60–70% (Vitolo et al., 2017). In current era, 18F-fluorodeoxyglucose positron emission tomography/computed tomography (^18^FDG PET/CT) is considered as standard-of-care for reliable staging and response assessment of aggressive malignant lymphomas, including DLBCL. Currently, ^18^FDG-PET/ CT at the end of treatment (ePET) is an accepted method for response assessment with ^18^FDG positivity is considered predictive of reduced survival in patients with malignant lymphoma (Cheson, 2011). There is large body of data favoring reliable role of ^18^FDG-PET during therapy (Interim-PET; iPET) for successful PET-guided treatment modification in Hodgkin lymphoma (Gallamini et al., 2018). But use of iPET for early response assessment and treatment modification in DLBCL remains controversial due to inconsistency in reported results (Dührsen et al., 2018; Yuan et al., 2019). Fundamental reason is low to modest positive predictive value (PPV) of iPET for DLBCL due to significantly high false positive (FP) results as it fails to discriminate between residual viable neoplastic tissue and a nonspecific inflammatory host response (Barrington et al., 2014). According to a recent meta-analysis, proportion of FP results for iPET are significantly higher (83%) than ePET (31.5%) (Adams and Kwee, 2016). While, negative predictive value (NPV) of iPET has been reported >80-85% in various reports with longer overall survival (OS) and progression free survival (PFS) (Pregno et al., 2012; Gallamini and Zwarthoed, 2017). But iPET is also known to be associated with false-negative (FN) findings as it can’t exclude clinically significant microscopic disease. A study reported a recurrence of 39.1% at a median follow-up of 30.8 months in DLBCL patients who had had a negative iPET (Kwon et al., 2016). Heterogeneity in results of various studies is caused by adjustable and non-modifiable factors seen in patient population of published studies. Adjustable factors include age and gender (significantly different age groups with gender predominance), non-standardized imaging protocols and interpretation criteria used in different studies. Non-modifiable factors include tumor behavior and presence of microenvironment cells like CD8+ tumor-infiltrating lymphocytes and PD1-positive lymphocytes (Fatima et al., 2019). So it is imperative to conduct studies upon patients’ population with minimal impact of above mentioned adjustable factors.

Aim of this study was to find progression free survival (PFS) and predictor of recurrence on follow-up ^18^FDG PET/CT in DLBCL patients who had a negative iPET using standardized imaging and reporting protocols.

## Materials and Methods

This prospective study was conducted at PET/CT Section of Department of Radiology, Aga Khan University Hospital Karachi, Pakistan from July 2017 till February 2020. Study was duly approved by ethical review committee of institute. We included patients with biopsy proven DLBCL who had ^18^FDG PET/CT studies at baseline and interim PET/CT (at least 10 days after 2nd or 4th chemotherapy) which was reported as complete metabolic response (CMR) using Deauville scoring system (score 1-3) (Meignan et al., 2017). All patients have received standard first line chemotherapy (CHOP with or without rituximab). We strictly followed a standardized protocol for ^18^FDG PET/CT as per European Association of Nuclear Medicine (EANM) guidelines for both studies (Boellaard et al., 2015). These patients were followed-up with ^18^FDG PET/CT performed at end of treatment or afterward for recurrence. Patients with Deauville score –X (indeterminate) on follow-up scans were either biopsied or followed with imaging at shorter intervals. Based on follow-up scan results, patients were categorized as having remission or sustained CMR (sCMR) or disease recurrence (DR). Kaplan Meier survival plot was used to estimate mean time of disease recurrence. Similarly, receiver operating characteristic (ROC) plot was used to see the impact of stage of disease, largest lesion size and highest SUVmax at baseline ^18^FDG PET/CT upon the disease recurrence. Progression free survival (PFS) was the period from iPET diagnosis until lymphoma progression, relapse after response, or death as a result of any cause (Cheson, 2011). 

^18^FDG PET/CT Imaging: ^18^FDG PET/CT was performed as per institutional protocol adopted from EANM guidelines (Boellaard et al., 2015). All patients had 4-6 hour fasting (only plain water was allowed) and a fasting blood sugar less than 200 mg% before receiving an intravenous ^18^FDG dose of 3 MBq/Kg in the uptake room. During uptake period (55 -75 minute) patients were requested to lie comfortably and allowed to take about 500-1,000 ml of plain water. Bladder was emptied prior to call the patient for PET/CT imaging suite equipped with Celesteion, Toshiba, Japan. A low dose CT examination (mid brain to mid-thigh) from head to toe followed by acquisition of PET imaging using 3 minute/bed position from toe to head in all patients. Follow up scans were performed using same protocols, keeping ^18^FDG dose, uptake time and hepatic SUVmean of baseline and follow-up studies within ± 10%, ± 15% and 20% minutes respectively as per published recommendations (Boellaard, 2011). 

Statistical Analysis: Comparisons between patient groups were performed using Student’s t test for continuous variables and the *χ*^2^ test for categorical variables. Continuous variables were described by mean ± standard deviation (SD). Receiver operating characteristic analysis was performed to calculate the area under the curve (AUC) and cut off values for age, stage, BMI, largest lesion size and highest SUVmax on baseline PET/CT with a corresponding 95% confidence interval as predictor(s) for tumor recurrence. Kaplan–Meier survival curve was plotted for recurrence free survival. Statistical significance was defined as P<0.05. Commercially available packages Microsoft excel 2010, Medcalc^®^ and statistical package for social sciences (SPSS 19^®^) were used.

## Results

**Figure 1:**
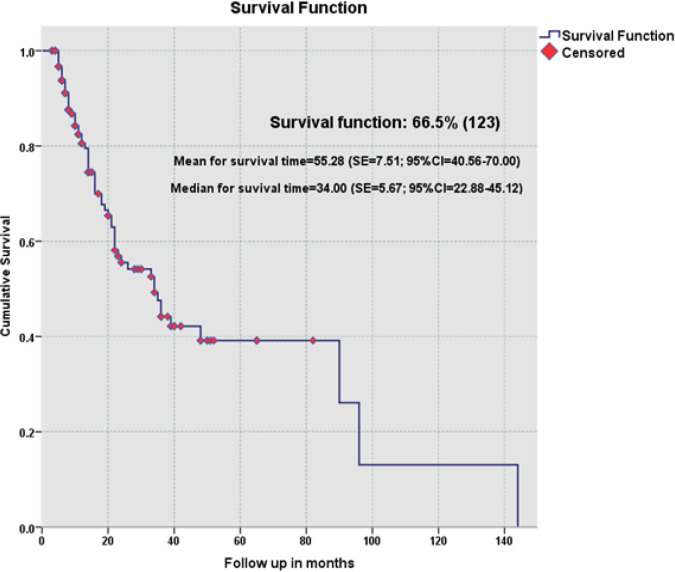
Kaplan Meier Survival Plot for Recurrence Free Survival of DLBCL Patients Based on Comlpete Metabolic Response on Follow up ^18^FDG PET/CT Studies. SE, Standard Error; CI, Confidence Interval

**Figure 2:**
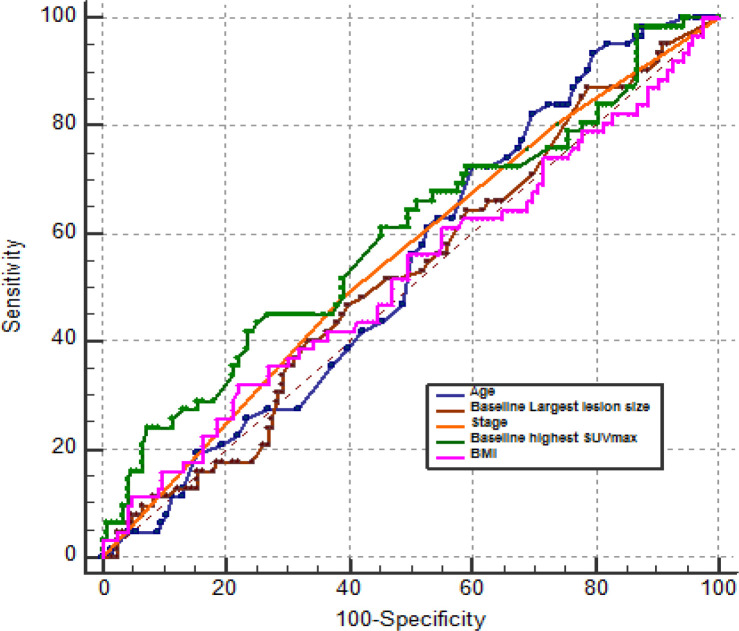
Receiver Operating Characteristics Plots for Predictors of Recurrence on Follow up ^18^FDG PET/CT Studies in DLBCL Patients with Complete Metabolic Response on iPET. SUV, Standardized uptake value; BMI, Body mass index

During study period, 185 patients with biopsy proven DLBCL who have achieved CMR (Deauville score: 1-3) on iPET were included. Based on follow-up (median: 11 ± 19 months) PET/CT findings, 123 patients (66%) remained in remission (sCMR) while 62 patients (34%) had developed metabolically active recurrence (DR) groups. No significant difference (p value >0.05) was seen in mean age (overall: 55 ±14 yr.), gender distribution (overall M;F: 63:37%), body mass index (overall: 26.47 ±4.96) and history of diabetes (overall: 36%) between sCMR and DR groups (Table 1). Similarly, no significant difference was observed for fasting blood sugar (overall: 109 ±34 mg%), ^18^FDG dose (overall: 171 ±37 MBq), uptake period (overall: 67 ±11 min) and mean hepatic uptake (overall: 1.76 ±0.47) between two groups ensuring strict adherence to standardized imaging protocol (Table 1). Furthermore, no significant difference was found for median baseline stage of disease (overall: stage 3 ±1), largest lesion on baseline PET/CT (overall: 64 ±49 mm), highest SUVmax on baseline scan (overall: 19 ±9.27) and median follow-up (overall: 11 ±19 months; range: 04-44 months) between sCMR and DR groups. The Kaplan Meier survival plot for time of recurrence revealed an overall mean PFS of 55.28 months (95% CI: 40.56 -70.0) (Figure 1). ROC analysis of various factors like age, stage of lymphoma, BMI, baseline largest lesion size and highest SUVmax, revealed that the highest SUVmax > 22.6 was the only significant predictor of disease recurrence (Figure 2 and Table 2).

**Table 1:** Demographic Comparison of DLBCL Patients with CMR on Interim and Follow-up 18FDG PET/CT Studies

Variables	Total N=185	sCMR N=123 (66%)	Disease Recurrence N=62 (34%)	Test/X^2^ values	*P*-values
Age Median ± SD	55 ± 14	55 ± 15	55 ± 12	0	1
(range)	(19-88) years	(19-88) years	(28-78) years		
BMI (Kg/m^2^) (Mean ± SD)	26.47 ± 4.96	26.14 ± 4.60	26.82 ± 5.42	0.893	0.373
Gender	117: 68	75:48:00	42:20:00	0.865	0.3523
(Male: Female)	(63 : 37%)	(61 : 39%)	(68 : 32%)		
Obesity (≥30Kg/m^2^)	39 (21%)	23 (19%)	16 (26%)	1.197	0.274
DM	36 (19%)	25 (20%)	11 (18%)	0.105	0.7457
FBS (mg/dl) (Mean ± SD)	109 ± 34	109 ± 33	111 ± 35	0.381	0.7035
FDG dose (MBq) (Mean ± SD)	171 ± 37	170 ± 36	175 ± 39	0.867	0.3871
Uptake period (Mean ± SD)	67 ± 11	70 ± 15	68 ± 10	-0.948	0.3442
Mean hepatic uptake (Mean ± SD)	1.76 ± 0.47	1.77 ± 0.47	1.73 ± 0.46	-0.55	0.5828
Highest SUVmax	19.00 ± 9.27	17.95 ± 8.08	21.00 ± 10.99	0.179	0.858
Mean ± SD (Range)	(3.9-61.2)	(3.7-47.6)	(3.9-61.2)		
Largest lesion	64 ± 49	64 ± 52	63 ± 50	-0.146	0.8841
Mean ± SD Range in mm	(05-266) mm	(05-266) mm	(09-237)		
Median Baseline stage	03 ± 01	03 ± 01	03 ± 01	0	1
Median follow up in months	11 ± 19 (04-144)	10 ± 16 (04-82)	14 ± 23 (05-144)	1.379	0.1697

*p<0.05; SD, Standard Deviation; BMI, Body Mass Index; DM, Diabetes Mellitus; FDG, Flurodeoxy glucose; FBS, Fasting Blood Sugar; SUV, Standardized Uptake Value; sCMR, sustained Complete Metabolic Response

**Table 2:** Receiver Operating Characteristics Analysis for Predictors of Recurrence on Follow up ^18^FDG PET/CT Studies in DLBCL Patients on Follow up

Variables	AUC	Criterion	Sensitivity	Specificity	SE	95% Confidence Internal		*P*- value
						lower limits	upper Limits	
Age (years)	0.541	>36	93.55	20.33	0.043	0.456	0.625	0.3442
Baseline largest lesion size	0.523	>23	87.1	21.14	0.045	0.436	0.611	0.6023
Baseline lymphoma Stage	0.554	>3	48.39	60.98	0.042	0.472	0.637	0.1965
Baseline highest SUVmax	0.595	>22.6	41.94	76.42	0.046	0.505	0.684	0.0375*
Baseline BMI	0.521	>29.675	32.26	78.05	0.0465	0.43	0.613	0.6471

*p<0.05; AUC, Area under Curve; SE, Standard Error; SUV, Standardized uptake value; BMI, Body mass index

## Discussion

^18^FDG-PET/CT is a widely used hybrid imaging modality for reliable staging of malignant lymphomas and response assessment as it can differentiate between viable tumor from post-treatment fibrosis and/or necrosis (Kitajima et al., 2019). Currently ^18^FDG-PET/CT performed at end of treatment (ePET) is recommended for response assessment in Hodgkin’s (HL) and NHL including DLBCL as positive scan has reasonably good predictive value for OS and PFS (Cheson, 2011). Based on published results, iPET has been considered good for response adapted management in HL (Gallamini et., 2018; André et al., 2017). However, the predictive value of iPET for response adapted treatment in DLBCL has been questioned due to inconsistent results (Adams and Kwee., 2016; Terasawa et al., 2009; Sun., 2015). Inhomogeneity in patients’ population, therapy regimens, imaging and reporting protocols have made it hard to clarify predictive accuracy of iPET in DLBCL (Burggraaff et al., 2019). We have tried our best to mitigate the impact of above mentioned confounding factors by strictly following standardized ^18^FDG PET/CT imaging protocol on same scanner and using Deauville 5-point scoring for reporting all studies. Furthermore, no significant difference in factors like age, gender predisposition, BMI, history of diabetes and stage of disease seem to have successfully mitigated their impact. However, this study has limitation in addressing non-modifiable factors like tumor behavior and presence of microenvironment cells like CD8+ tumor-infiltrating lymphocytes and PD1-positive lymphocytes (Fatima et al., 2019). Regarding the treatment regimens, all of studied patients have had received CHOP or R-CHOP till iPET was done which again increases the statistical strength of our study. 

As a matter of fact, most of the published studies have addressed the predictive value of positive iPET in DLBCL with unsatisfactory sensitivity and specificity (Milana et., 2015). In this study we tried to determine how many patients would have recurrence after achieving a complete remission as documented by a negative iPET (DS ≤3). Despite of variable results, NPV of iPET in DLBCL has been reported greater than 80% (Moskowitz and Shoder., 2015). However, in our study, NPV of iPET was found to be 66% which is significantly lower. On reviewing literature, Jerusalem et al., (2000) also reported a NPV of 67% in a small study of 28 patients including 16 with DLBCL. Another study published in 2005 upon 90 DLBCL patients revealed a NPV of 70% at 24 months median follow-up (Haioun et al., 2005). Therefore plausible explanations could be a relatively homogenous patient population and use of a standardized imaging and reporting criteria compared with published studies. 

The reported recurrence after completion of treatment in patients with DLBCL is around 30-40% (Pfreundschuh et al., 2011). In our study, 62/185 patients with negative iPET developed disease recurrence during a median follow-up of 11 months (false negative: 34%). This recurrence rate is significantly higher than published studies. Carr et al., (2014) reported a recurrence of 10% in a large cohort of patients with negative iPET. Similarly study by Mamot et al., (2015) reported a recurrence of 24% in 55 patients with negative iPET. However, recurrence in our study is in concordance with the study published by Kwon et al., (2016) upon 92 DLBCL patients with negative iPET who experienced recurrence during a median follow-up of 30.8 months giving 39.1% false negative findings. These different recurrence rates in various studies are difficult to explain but warn us that a negative iPET does not guarantee no-recurrence in DLBCL patients. A possible reason for false negative iPET could be stunning of glucose metabolism by chemotherapeutic agent(s) which likely to happen in first 10 days post-treatment (Engles et al., 2006). However, the odds of metabolic stunning is less likely in our study because as per departmental protocol iPET was performed at least 10 days after recent chemotherapy. So the most plausible explanation for recurrence in negative iPET could be the presence of clinically significant viable tumor cells in non-avid residual mass(s) (less than liver SUVmax) which were beyond the spatial resolution of our scanner. This high recurrence with negative iPET indeed questions the diagnostic accuracy of Deauville scores 1-3 (no uptake or ≤ mediastinal or ≤ liver uptake) to interpret an iPET as CMR. Recently published results from Positron Emission Tomography-guided Therapy of Aggressive non-Hodgkin Lymphomas (PETAL Trial) found better diagnostic accuracy of delta SUVmax (∆SUVmax ≤66%) than Deauville scoring >3 on iPET for dose intensification to avoid over-treatment (Rekowski et al., 2020). However, DS ≤3 and ∆SUVmax ≤66% were found to have comparable event free survival (EFS) in PETAL trial (Rekowski et al., 2020). Kwonet al., (2016) used DS-1 for negative iPET in homogenous population but the relapse rate is similar to our study with DS ≤ 3 as negative iPET in homogenous population with standardized imaging and reporting protocols. But they had significantly longer median follow-up (30.8 months) than current study (11 months).

In this study, only the highest SUVmax (cut-off > 22.6) in baseline ^18^FDG PET/CT was found an independent predictor for PFS in patients with a negative iPET. Prognostic significance of pretreatment SUVmax of ^18^FDG PET/CT has been explored by various studies with variable inferences. Study by Chihara et al., (2011) on 110 patents found 3-year PFS rates in patients with baseline SUV < 30 and those with SUV ≥ 30 were 78 and 51%, respectively. Another study published in 2016 found pretreatment SUVmax > 10.5 as a significant predictor for PFS only on univariate analysis (Kwon et al., 2016). Park et al., (2012) also reported baseline SUVmax as predictor of PFS in patients with DLBCL. However, Kim et al., (2013) found total lesion glycolysis (TLG) as a better prognostic indicator of PFS than SUVmax and international prognostic index (IPI). 

This study has some limitations. Firstly, we did not mention established prognostic factors like serum lactic dehydrogenase (LDH) and IPI which will be correlated with outcome in future study. Secondly, we did not use TLG, another semiquantitative parameter, which is known to be less affected by some imaging and non-imaging parameters than SUVmax. However, it also a known fact that SUVmax is the most common parameter use in clinical practice and estimation of TLG is time consuming in imaging systems not having a software option which was the case with our facility. Thirdly, we did not compare positive iPET with negative. However, as mentioned earlier, positive predictive value of iPET in DLBCL has extensively been studied and we find fewer studies regarding the relapse rate in negative iPET. Fourth, the standard regimen in this study was CHOP with or without rituximab. We understand this is an important limitation as rituximab is prone to induce false positive ^18^FDG uptake. But this would have led to false positive iPET while our study was focused over negative iPET. 

We conclude that recurrence is found in 34% of DLBCL patients with a negative interim ^18^FDG PET/CT using standardized imaging and reporting protocols. Despite of early response, these patients need continued intensive follow-up especially those with a baseline SUVmax > 22.6.

## Competing of Interest

The authors don’t have any financial and/or Institutional conflict of interest to declare.

## References

[B1] Adams HJ, Kwee TC (2016). Proportion of false-positive lesions at interim and end-of-treatment FDG-PET in lymphoma as determined by histology: systematic review and meta-analysis. Eur J Radiol.

[B2] Adams HJ, Kwee TC (2016). Prognostic value of interim FDG-PET in RCHOP-treated diffuse large B-cell lymphoma: systematic review and meta-analysis. Crit Rev Oncol Hematol.

[B3] André MPE, Girinsky T, Federico M (2017). Early positron emission tomography response-adapted treatment in stage I and II Hodgkin lymphoma: final results of the randomized EORTC/LYSA/FIL H10 trial. J Clin Oncol.

[B4] Barrington SF, Mikhaeel NG, Kostakoglu L (2014). Role of imaging in the staging and response assessment of lymphoma: consensus of the International Conference on Malignant Lymphomas Imaging Working Group. J Clin Oncol.

[B5] Boellaard R (2011). Need for standardization of 18F-FDG PET/CT for treatment response assessments. J Nucl Med.

[B6] Boellaard R, Bolton RD, Oyen WJ (2015). FDG PET/CT: EANM procedure guidelines for tumour imaging: version 20. Eur J Nucl Med Mol Imag.

[B7] Burggraaff CN, Jong A, Hoekstra OS (2019). Predictive value of interim positron emission tomography in diffuse large B-cell lymphoma: a systematic review and meta-analysis. Eur J Nucl Med Mol Imag.

[B8] Carr R, Fanti S, Paez D (2014). Prospective international cohort study demonstrates inability of interim PET to predict treatment failure in diffuse large B-cell lymphoma. J Nucl Med.

[B9] Cheson BD (2011). Role of functional imaging in the management of lymphoma. J Clin Oncol.

[B10] Chihara D, Oki Y, Onoda H (2011). High maximum standard uptake value (SUVmax) on PET scan is associated with shorter survival in patients with diffuse large B cell lymphoma. Int J Hematol.

[B11] Dührsen U, Müller S, Hertenstein B (2018). Hüttmann A1 PETAL Trial Investigators Positron emission tomography-guided therapy of aggressive non-Hodgkin lymphomas (PETAL): A multicenter, randomized phase III trial. J Clin Oncol.

[B12] Engles JM, Quarless SA, Mambo E (2006). Stunning and its effect on 3HFDG uptake and key gene expression in breast cancer cells undergoing chemotherapy. J Nucl Med.

[B13] Fatima N, Zaman MU, Zaman A (2019). Predictors of metabolic response in propensity-matched lymphoma patients on interim 18F-fluorodeoxyglucose positron-emission tomography/computed tomography using standardized imaging and reporting protocol: Do we really have one?. World J Nucl Med.

[B14] Gallamini A, Zwarthoed C (2017). Interim FDG-PET Imaging in Lymphoma. Semin Nucl Med.

[B15] Gallamini A, Tarella C, Viviani S (2018). Early chemotherapy intensification with escalated BEACOPP in patients with advanced-stage Hodgkin lymphoma with a positive interim positron emission tomography/computed tomography scan after two ABVD cycles: long-term results of the GITIL/FIL HD 0607 Trial. J Clin Oncol.

[B16] Gisselbrecht C, Glass B, Mounier N (2010). Salvage regimens with autologous transplantation for relapsed large B-cell lymphoma in the rituximab era. J Clin Oncol.

[B17] Haioun C, Itti E, Rahmouni A (2005). [18F] fluoro- 2-deoxy-D-glucose positron emission tomography (FDG-PET) in aggressive lymphoma: an early prognostic tool for predicting patient outcome. Blood.

[B18] Jerusalem G, Beguin Y, Fassotte MF, Najjar F, Paulus P (2000). Persistent tumor 18F-FDG uptake after a few cycles of polychemotherapy is predictive of treatment failure in non-Hodgkin’s lymphoma. Haematologica.

[B19] Kim TM, Paeng JC, Chun IK (2013). Total lesion glycolysis in positron emission tomography is a better predictor of outcome than the International Prognostic Index for patients with diffuse large B cell lymphoma. Cancer.

[B20] Kitajima K, Okada M, Yoshihara K (2019). Predictive value of interim FDG-PET/CT findings in patients with diffuse large B-cell lymphoma treated with R-CHOP. Oncotarget.

[B21] Kwon SH, Kang DR, Kim J (2016). Prognostic value of negative interim 2-[¹8F]-fluoro-2-deoxy-d-glucose PET/CT in diffuse large B-cell lymphoma. Clin Radiol.

[B22] Mamot C, Klingbiel D, Hitz F (2015). Final results of a prospective evaluation of the predictive value of interim positron emission tomography in patients with diffuse large B-cell lymphoma treated with R-CHOP-14 (SAKK 38/07). J Clin Oncol.

[B23] Meignan M, Gallamini A, Haioun C 2009): Report on the first International Workshop on Interim-PET-scan in lymphoma. Leuk Lymphoma.

[B24] Milana M, Marko E, Miroslav, Dugandzija T (2015). Importance of PET/CT scan use in planning radiation therapy for lymphoma. Asian Pac J Cancer Prev.

[B25] Moskowitz C, Shoder H (2015). Current status of the role of PET imaging in diffuse large B-cell Lymphoma. Semin Hematol.

[B26] Park S, Moon SH, Park LC (2012). The impact of baseline and interim PET/CT parameters on clinical outcome in patients with diffuse large B cell lymphoma. Am J Hematol.

[B27] Pfreundschuh M, Kuhnt E, Trumper L (2011). CHOP-like chemotherapy with or without rituximab in young patients with good-prognosis diffuse large-B-cell lymphoma: 6-year results of an open-label randomized study of the MabThera International Trial (MInT) Group. Lancet Oncol.

[B28] Pregno P, Chiappella A, Bello` M (2012). Interim18-FDG-PET/CT failed to predict the outcome in diffuse large B-cell lymphoma patients treated at the diagnosis with rituximab-CHOP. Blood.

[B29] Rekowski J, Hüttmann A, Schmitz C (2020). Interim PET evaluation in diffuse large B-cell lymphoma employing published recommendations: Comparison of the Deauville 5-point scale and the ΔSUVmax method. J Nucl Med (published.

[B30] Sun N, Zhao J, Qiao W, Wang T (2015). Predictive value of interim PET/CT in DLBCL treated with R-CHOP: meta-analysis. Biomed Res Int.

[B31] Terasawa T, Lau J, Bardet S (2009). Fluorine-18-fluorodeoxyglucose positron emission tomography for interim response assessment of advanced-stage Hodgkin’s lymphoma and diffuse large B-cell lymphoma: a systematic review. J Clin Oncol.

[B32] Vitolo U, Trněný M, Belada D (2017). Obinutuzumab or rituximab plus cyclophosphamide, doxorubicin, vincristine, and prednisone in previously untreated diffuse large B-cell lymphoma. J Clin Oncol.

[B33] Yuan L, Kreissl MC, Su L (2019). Prognostic analysis of interim 18F-FDG PET/CT in patients with diffuse large B cell lymphoma after one cycle versus two cycles of chemotherapy. Eur J Nucl Med Mol Imag.

